# Diagnostic and Phylogenetic Insights into a Human Rabies Virus Isolate from Romania

**DOI:** 10.3390/v18040475

**Published:** 2026-04-17

**Authors:** Vlad Vuta, Maria Gradinaru, Mihnea Hurmuzache, Florica Bărbuceanu, Lenuta Zamfir, Răzvan Moțiu, Laura Schmid, Dirk Höper, Sten Calvelage, Thomas Müller, Conrad M. Freuling

**Affiliations:** 1 National Reference Laboratory for Animal Diagnosis and Health, Institute for Diagnosis and Animal Health, 050557 Bucharest, Romania; vlad.vuta@idah.ro (V.V.); florica.barbuceanu@idah.ro (F.B.); lenuta.zamfir@idah.ro (L.Z.); razvan.motiu@idah.ro (R.M.); 2 Clinical Hospital of Infectious Diseases, 700116 Iasi, Romania; mariuca777@gmail.com (M.G.); mihneaeudoxiu.hurmuzache@gmail.com (M.H.); 3 Faculty of Veterinary Medicine, 050097 Bucharest, Romania; 4 Friedrich-Loeffler-Institute (FLI), Insel Riems, 17493 Greifswald, Germany; laura.schmid@fli.de (L.S.); dirk.hoeper@fli.de (D.H.); sten.calvelage@fli.de (S.C.); thomas.mueller@fli.de (T.M.)

**Keywords:** rabies, human case, Romania, molecular epidemiology, RT-PCR, next-generation sequencing

## Abstract

Rabies is a fatal zoonotic disease once clinical symptoms develop. In Europe, sustained animal rabies control programs have led to a marked decline in animal rabies and subsequently human rabies cases; however, sporadic infections continue to occur. In July 2025, a fatal case of autochthonous (locally acquired) human rabies was confirmed in Romania following a stray dog bite in a patient who did not receive post-exposure prophylaxis (PEP). Here, we report the first molecular characterization of a human rabies virus (RABV) strain isolated in Romania and place it in the context of contemporaneously circulating animal-derived RABV strains. Rabies virus infection was confirmed intra vitam by fluorescent antibody testing and both conventional and real-time RT-PCR on cerebrospinal fluid and saliva, with postmortem confirmation on skin and brain tissue. Whole-genome sequencing was performed on the human isolate and on 22 animal-derived RABV strains collected in northern Romania in 2025. Phylogenetic analyses revealed that all recent Romanian sequences clustered within the North-East European (NEE) rabies virus phylogenetic group and segregated into two geographically distinct genetic clusters: a north-western cluster, closely related to strains from Slovakia and Poland, and a larger north-eastern cluster, linked to viruses circulating in eastern Romania and the Republic of Moldova. The human-derived RABV genome was grouped within the north-eastern cluster and showed the highest genetic similarity to animal viral strains from the same geographical area, supporting a local transmission event. This demonstrates the importance of integrating human viral genomic data into the national rabies surveillance framework.

## 1. Introduction

Rabies is an acute viral zoonosis caused by lyssaviruses, including the prototypical rabies virus (RABV), a member of the genus *Lyssavirus* within the Rhabdoviridae family [1]. Despite the availability of effective vaccines and immunoglobulins, rabies remains one of the deadliest infectious diseases, with an almost 100% case-fatality rate once clinical symptoms develop [2]. Human infection occurs predominantly through the bites of infected animals, with domestic dogs globally representing the main transmitter and reservoir. In Europe, wildlife species, particularly the red fox (*Vulpes vulpes*), have historically played a role as a reservoir [3]. Over the past decades, coordinated rabies control efforts, predominantly focused on oral rabies vaccination (ORV) campaigns targeting fox populations, have substantially reduced animal rabies incidence or even eliminated rabies across many European countries [4,5,6], also leading to a corresponding decline of human rabies cases [7].

Romania has historically been endemic for rabies in both wild and domestic animals, with fox-mediated sylvatic transmission predominating [8]. Molecular epidemiological studies demonstrated considerable genetic heterogeneity among Romanian RABV strains, reflecting the country’s geographical position at the crossroads of Central and Eastern Europe and the circulation of distinct genetic lineages [9]. Although ORV programs had reduced the number of reported animal cases, raising the expectation to control rabies within the European Union (including Romania) [10], RABV circulation persists in certain regions, particularly in northern and eastern Romania [7]. Human rabies cases in Romania have become rare; however, when they occur, they are often associated with exposure to wildlife, pet animals or stray dogs and delayed or absent post-exposure prophylaxis (PEP) [11].

Laboratory confirmation of rabies in living patients remains a challenge due to intermittent viral shedding and low viral loads in accessible clinical samples [2]. Nonetheless, advances in molecular diagnostic techniques, like established conventional and real-time RT-PCR protocols, have improved the sensitivity of intra vitam diagnosis using sample matrices such as saliva, cerebrospinal fluid, and skin biopsies [12]. In parallel, next-generation sequencing (NGS) has become an essential tool for molecular epidemiology, enabling high-resolution analysis of RABV transmission dynamics and its transboundary spread [13]. To date, no molecular characterization of a human-derived RABV strain has been reported from Romania. The absence of such data represents a gap in national rabies surveillance and limits the integration of human cases into phylogenetic and phylogeographic analyses. In this study, we present the first phylogenetic characterization of a human RABV isolate from Romania and compare it with currently circulating animal-derived RABV isolates from Romania.

## 2. Materials and Methods

### 2.1. Human Case and Samples

In June 2025, the Institute for Diagnosis and Animal Health (IDAH), Bucharest, received cerebrospinal fluid (CSF) and saliva samples collected from a 45-year-old male patient from Voinești, Iași County, Romania. At the end of February 2025, the patient had sustained a bite injury to the left hand inflicted by an unidentified dog that had entered the household. The patient subsequently presented to a primary care physician, where initial wound management was performed and antibiotic therapy was initiated. In addition, the patient was referred for rabies post-exposure prophylaxis (PEP) but this was declined by the patient [14]. In mid-June 2025, the patient developed psychomotor agitation accompanied by hallucinations and, in the context of ethanol consumption, was admitted to a psychiatric service. During a 5-day hospitalization, treatment with antipsychotic agents led to partial improvement of psychomotor agitation; however, the patient progressively developed headache, gait instability, and hypersalivation. On days 5–6 following the onset of neuropsychiatric symptoms, a diagnosis of encephalitis was established, with cerebrospinal fluid (CSF) analysis revealing 19 cells/mm^3^. The patient was subsequently transferred, under orotracheal intubation, to the Intensive Care Unit (ICU) of the Infectious Diseases Hospital in Iași. Rabies was clinically suspected following the onset of neurological symptoms. CSF and saliva samples were collected and sent to the National Reference Laboratory for Animal Diagnosis and Health (IDAH). Under advanced supportive care in the ICU, the patient survived for 23 days; death occurred on 13 July 2025, approximately one month after symptom onset. Postmortem brain and skin biopsy samples were also submitted to IDAH for confirmatory testing.

To contextualize the human-derived RABV, 22 rabies-positive brains from domestic and wild animals (fox, jackal, dog, cattle, cat, badger) originating from those five northern Romanian counties (Iași, Maramureș, Vaslui, Satu Mare, and Botoșani) with rabies presence in 2025 were included in the study. The dataset represents a selected subset of cases, chosen to reflect both spatial heterogeneity across the affected region and diversity in animal species.

### 2.2. Diagnostic Testing

Intra vitam and post-mortem rabies diagnosis in the human patient consisted of conventional RT-PCR and real-time RT-PCR using previously published primers and protocols [15,16]. Briefly, RNA extracted from clinical samples and brain homogenates using the Total RNA purification Kit (Jena Bioscience, Jena, Germany) was added to master mixes for conventional RT-PCR [15] and real-time RT-PCR [16] using the One-step RT-PCR kit (QIAGEN, Hilden, Germany) or the QuantiTect^®^ SYBR^®^ Green RT-PCR kit (QIAGEN, Hilden, Germany), respectively. Post-mortem confirmation was performed using the direct fluorescent antibody test (FAT) on brain and skin tissue according to standard protocols [17] with the fluorescein isothiocyanate (FITC)-labeled anti-rabies monoclonal globulins from Fujirebio (Tokyo, Japan) and sifin (Berlin, Germany). The image was taken with a microscope (ECLIPSE Ti-S, NIKON, Tokyo, Japan) at 10× magnification.

### 2.3. Tissue Preparation, Immunolabelling and Confocal Laser Scanning Microscopy (CLSM)

Cerebral tissue preparation and immunolabelling were performed as previously described [18], with minor modifications (See Supplementary Material, [1,2]). Rabies virus phosphoprotein (RABV P) was detected using a polyclonal rabbit serum and polyclonal rabbit anti-N serum N161/5 [19] followed by Alexa Fluor-conjugated secondary antibodies. Image stacks were acquired with a confocal microscope (Stellaris 8, Leica, Wetzlar, Germany) and processed using ImageJ software (1.54f, https://imagej.net/).

### 2.4. Next-Generation Sequencing

Total RNA extracted from brain tissue was used for double-stranded cDNA synthesis, library preparation, and sequencing on an Ion Torrent™ S5XL platform (Life Technologies, Darmstadt, Germany) as published [20]. Sequencing libraries were quality controlled prior to sequencing (See Supplementary Material, [19,21,22,23,24,25,26,27,28]).

### 2.5. Genome Assembly and Phylogenetic Analysis

Raw sequencing reads were mapped against a reference RABV genome, and consensus sequences were generated following quality control. Complete genomes were aligned with publicly available Eastern European RABV sequences using MAFFT (version 7). Phylogenetic analysis was conducted using IQ-TREE with enabled model selection and 100,000 ultrafast bootstrap replicates. Tree visualization was performed using iTOL. Generated complete/nearly complete genome sequences are accessible at the European Nucleotide Archive (ENA) under the project number PRJEB108651.

## 3. Results

### Laboratory Confirmation of Human Rabies

Rabies virus antigen was detected by FAT in skin and brain tissue samples from the patient. Viral RNA was detected in saliva and CSF by both conventional and real-time RT-PCR, with the lowest Ct value observed in brain tissue (Table 1).

**Figure 1:**
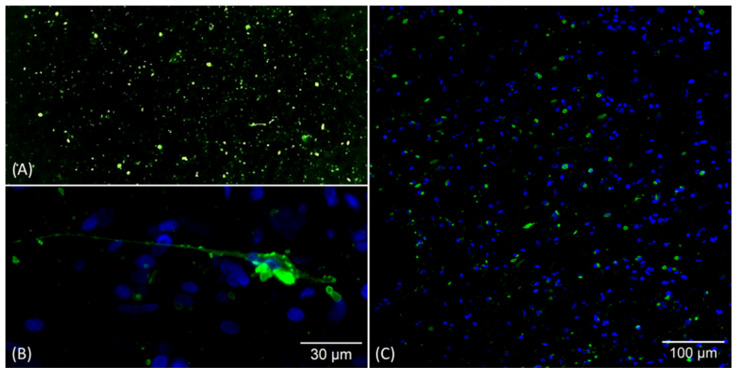
(**A**) Apple-green immunofluorescence of the rabies virus nucleoprotein in a brain smear for the fluorescent antibody test (FAT) using the sifin conjugate at 10× magnification. (**B**) Confocal laser scanning microscopy (CLSM): Cerebral tissue maximum z-projection of 50 optical slices acquired with a step size of 0.3 µm; green = RABV P; blue = cell nuclei. (**C**) CLSM: Cerebral tissue; green = RABV-P; blue = cell nuclei.

Confocal microscopy of immunolabeled brain sections revealed characteristic apple-green fluorescent viral inclusions within neuronal tissue, consistent with RABV infection. Notably, while high-resolution imaging of RABV-infected brains demonstrates viral antigen in neuronal structures [29], e.g., axons, the absence of the latter suggests a disintegration of the structures within the central nervous system (Figure 1).

**Figure 2:**
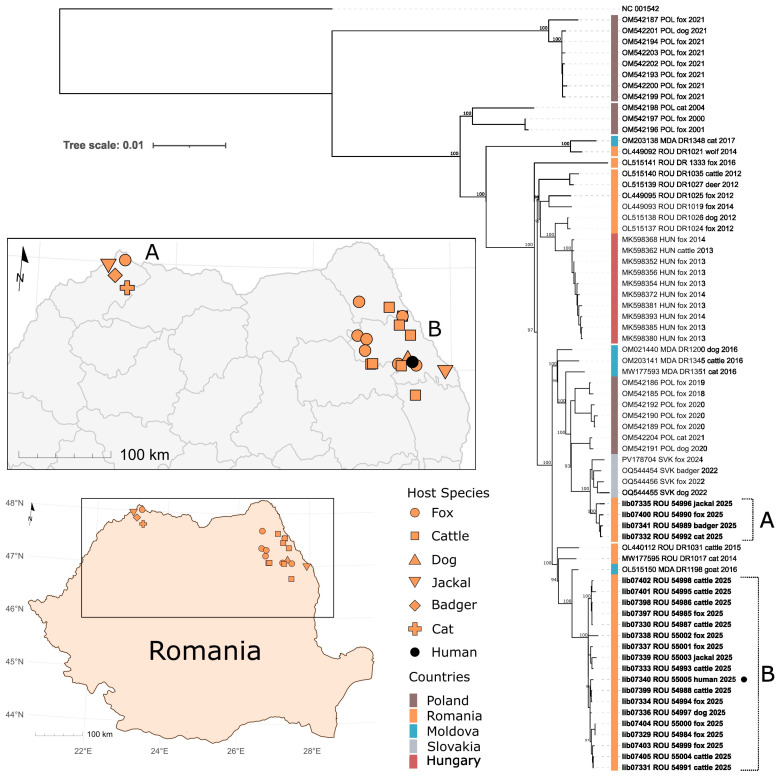
Geographic distribution and phylogenetic relationship of investigated Romanian RABV cases from 2025. Newly generated virus sequences are highlighted in bold and the spillover into humans is marked by a black dot in the Maximum Likelihood phylogenetic tree. Two genetically distinct groups of Romanian RABV were identified among cases from 2025 that correlate with the geographic separation of cases in a northwest (**A**) and north-east outbreak group (**B**). Maximum Likelihood phylogenetic tree construction was realized with IQTree (best fit model according to ModelFinder: TVM + F + G4) and maps were created with R and combined with the phylogenetic tree using Inkscape (Version 1.4, https://inkscape.org).

Whole-genome sequencing generated 22 complete RABV genomes ranging from 11,862 to 11,891 nucleotides with mean coverage depths between 95 and 2888 reads per base (Table 2). Phylogenetic analysis, including sequences from Poland, Slovakia, Moldova, Hungary, and previously generated RABV genomes from Romania (Table S1), showed that all Romanian sequences clustered within the North-East European (NEE) phylogenetic group. Moreover, the resulting tree revealed 2 distinct genetic groups formed by the new Romanian sequences that mirror the geographical segregation of the investigated cases in a northwest (Figure 2A) and a north-east outbreak cluster (Figure 2B). Sequences of group A (n = 4) are closest related to RABV sequences from Slovakia and Poland while members of group B (n = 18) cluster with 1 Moldovan sequence from 2016 and 2 Romanian cases from the east/north-east located counties Galați (OL440112) and Vaslui (MW177595). The sequence of the reported human spillover (Figure 2, lib07340, black dot) shows a close genetic relationship with samples from group B which is formed by RABV-confirmed cases from domestic (cattle and dogs) and wildlife animals (jackal and fox). In detail, the closest related RABV sequences were described for cases reported in near geographic proximity to the human case, namely 54988 (lib7399, cattle) and 54994 (lib7334, fox), which further emphasizes the correlation between the genetic relationship and the geographic distribution of the investigated RABV strains.

## 4. Discussion

This study presents the first molecular characterization of a human-isolated rabies virus strain from Romania and provides genomic evidence linking a fatal human rabies infection to locally circulating animal RABV strains. Although the incidence of rabies in Europe has decreased significantly due to sustained animal control measures, this case highlights that rabies remains a preventable but persistent public health threat when post-exposure prophylaxis (PEP) is not administered. In this case, the lack of PEP following a stray dog bite was a critical factor contributing to the fatal outcome, underscoring that awareness, access to healthcare, and adherence to prophylactic guidelines remain essential components of rabies prevention.

From a laboratory diagnostic perspective, the successful intra vitam detection of rabies virus RNA and antigen emphasizes the importance of molecular diagnostic capacity and close collaboration between clinicians and reference laboratories, particularly in countries where human rabies cases are rare and clinical awareness may be low. Rabies virus RNA levels in infected brain tissue are typically high, with real-time RT-PCR assays commonly yielding Ct/Cq values in the range of approximately 15–25 (e.g., Table 2) [16,30,31]. In this case, a ct value of 11 in brain material indicates an extremely high viral load.

Molecular and phylogenetic analyses provide important insights into RABV circulation in Romania. All investigated strains exclusively clustered with sequences previously assigned to one lineage, namely RO#6, within the North-East European (NEE) phylogenetic group [9,26]. Interestingly, this is a distinct change from the presence of six different RABV lineages detected in three phylogenetic groups between 2005 and 2008 when rabies was present within the entire country [9]. However, the apparent decrease in genetic diversity is, to some extent, contradicted by the formation of distinct clusters within the RO#6 lineage, as demonstrated by the two clusters corresponding to the north-western and north-eastern regions that highlight the ongoing geographical structuring of RABV circulation in the country. On the basis of full genomes, the north-western cluster showed close genetic relationships with strains from Slovakia and Poland, while the larger north-eastern cluster was linked to viral strains circulating in eastern Romania and the Republic of Moldova, suggesting transboundary virus transmission dynamics [32]. In fact, partial N- and G- gene sequence analyses suggest that this diversity is mirrored by the presence of these genetic variants in the Ukraine [33].

It is worth noting that the human-isolated RABV strain belongs to the North-East European (NEE) group and exhibits the highest genetic similarity to animal strains from the same geographical area. This provides molecular evidence for a local transmission event involving both wildlife and domestic animals. The absence of a rabies virus isolate from a dog with 100% sequence identity to the patient suggests that current animal rabies surveillance does not capture all circulating infections, especially in settings with endemic transmission and free-roaming dogs or wildlife reservoirs. Surveillance systems in such contexts are often limited by low detection probabilities and incomplete case ascertainment [34]. An alternative explanation of the observed missing identical sequences is based on an intra-host evolution event in the human host. However, with the lack of sequences from the original infectious agent present in the inoculum (i.e., the saliva of the biting dog), the question of whether the virus accumulated further mutations during infection cannot be answered by this study.

**Figure 3:**
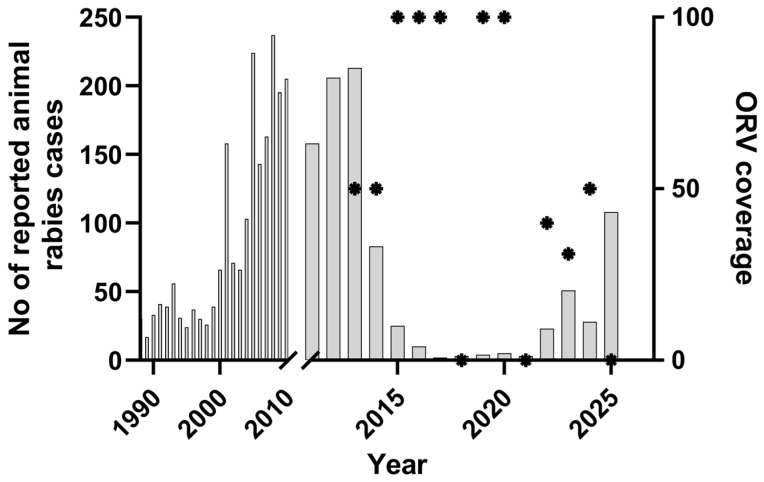
Graph showing the annual number of Romanian animal rabies cases reported to the Rabies Bulletin Europe database (bars). From 2013 onwards, the percentage coverage of oral rabies vaccination campaigns is indicated (*, right y-axis): 100% is the entire country covered by two campaigns, one in spring and one in autumn. ORV data sources: [35,36,37].

These findings reinforce the importance of integrated “One Health” surveillance approaches, which combine molecular data from both human and animal cases to better understand transmission pathways and coordinate control and eradication strategies [13]. The initial success of rabies control in Romania, with few reported cases (Figure 3) from 2017 to 2021 originating from areas close to the border, was based on ORV campaigns targeting foxes (source: Rabies Bulletin Europe database).

However, the interruption of oral rabies vaccination (ORV) campaigns targeting foxes [36]—with multiple seasonal baiting rounds missed—has been associated with a resurgence of rabies in both wild and domestic animals in Romania. Sustained, twice-annual ORV over consecutive years is essential to break transmission and maintain population immunity in reservoir species, and interruptions in such programs are known to compromise control efforts and can lead to renewed viral circulation in endemic areas. Between January and December 2025, 108 rabies cases were identified (Figure 3). The continued presence of rabies in the animal reservoir poses a direct risk to humans. This human case furthermore underscores the importance of stray dogs as a source of human exposure. High densities of stray and free-roaming dogs [38] and frequent interactions with humans, particularly in peri-urban and rural settings, maintain the risk of rabies transmission even in regions where wildlife rabies has declined.

In conclusion, this study provides the first genomic evidence corroborating a human RABV infection in Romania linked to animal transmission and demonstrates the importance of molecular characterization of human cases for national surveillance [14].

The risk for rabies transmission can be effectively minimized by conducting ORV campaigns following international guidelines [39]. Also, strengthening rabies awareness, ensuring rapid access to PEP, maintaining diagnostic techniques, and continuing coordinated animal control measures are critical steps toward eliminating human rabies in Romania and eventually becoming a rabies-free region.

## Figures and Tables

**Table 1:** Results of RT-PCR on intra vitam and post-mortem samples using the Heaton [15] and Wakeley protocols [16].

Specimen	Sampling	RT-PCR	RT-qPCR
Saliva	intra vitam	Pos	21.6
CSF *	intra vitam	Pos	28.5
Skin	post-mortem	Pos	18.4
Brain	post-mortem	Pos	11.9

* CSF = cerebrospinal fluid.

**Table 2:** Metadata and sequencing results of 22 Romanian RABV cases from 2025.

Sample		Metadata						Laboratory Results				
Sample ID	Library ID	Host Species	Sample	Date	Latitude	Longitude	County	Ct Value (RT-qPCR)	Reads Total	Reads RABV	Virus Reads [%]	Mean Coverage [Reads per Base]
54984	7329	Fox	Brain	5 March 2025	47.343215	26.652739	Iași	17.6	3,623,019	10,231	0.28	300
54987	7330	Cattle	Brain	26 February 2025	47.5342	27.3027	Iași	20.52	3,420,940	19,978	0.58	582
54991	7331	Cattle	Brain	9 May 2025	47.06617	26.84550	Iași	21.16	2,569,010	4690	0.18	135
54992	7332	Cat	Brain	8 May 2025	47.7477	23.2852	Maramureș	19.1	3,241,499	6247	0.19	179
54993	7333	Cattle	Brain	25 April 2025	46.76159	27.48180	Vaslui	20.81	3,056,978	28,050	0.92	787
54994	7334	Fox	Brain	17 April 2025	47.068648	27.237996	Iași	17.23	3,009,007	6496	0.22	187
54996	7335	Jackal	Brain	9 April 2025	47.97980	23.01362	Satu Mare	17.2	3,098,484	9532	0.31	273
54997	7336	Dog	Brain	31 March 2025	47.10900	27.37544	Iași	17.84	6,910,564	3312	0.05	95
55001	7337	Fox	Brain	21 July 2025	47.053201	27.495400	Iași	17.63	3,542,508	33,980	0.96	978
55002	7338	Fox	Brain	23 July 2025	47.674978	26.672376	Botoșani	16.05	3,127,490	8002	0.26	335
55003	7339	Jackal	Brain	23 July 2025	47.005184	27.911642	Iași	17.55	3,352,153	4918	0.15	143
55005	7340	Human	Brain	17 July 2025	47.072043	27.439271	Iași	17.09	2,968,451	39,236	1.32	333
54989	7341	Badger	Brain	15 January 2025	47.86899	23.11980	Satu Mare	16.63	2,760,060	10,373	0.38	293
54985	7397	Fox	Brain	3 March 2025	47.5332	27.3011	Iași	17.66	2,680,181	9897	0.37	291
54986	7398	Cattle	Brain	28 February 2025	47.34768	27.41507	Iași	20.43	2,626,135	8569	0.33	248
54988	7399	Cattle	Brain	21 February 2025	47.04906	27.28104	Iași	20.68	5,615,976	41,215	0.73	1.159
54990	7400	Fox	Brain	9 January 2025	48.02294	23.25541	Satu Mare	18.76	3,363,087	7471	0.22	215
54995	7401	Cattle	Brain	14 April 2025	47.62177	27.10982	Botoșani	18.67	3,502,619	32,828	0.94	909
54998	7402	Cattle	Brain	18 March 2025	47.446753	27.255463	Iași	17.83	3,513,423	7385	0.21	213
54999	7403	Fox	Brain	17 March 2025	47.309757	26.782966	Iași	15.79	5,024,369	20,166	0.40	566
55000	7404	Fox	Brain	10 March 2025	47.197880	26.761450	Iași	16.39	2,732,891	6868	0.25	199
55004	7405	Cattle	Brain	23 July 2025	47.07065	26.871912	Iași	17.63	3,340,214	105,518	3.16	2.888

## Data Availability

Sequencing data presented in the study are openly available in the European Nucleotide Archive (ENA), under the project number PRJEB108651.

## Supplementary Materials

The following supporting information can be downloaded at: https://www.mdpi.com/article/10.3390/v18040475/s1, Methods S1: Next-generation sequencing (NGS); Tissue preparation and immunolabelling; Confocal laser scanning microscopy and image processing. Table S1: Reference sequences selected from public databases for the genetic classification of Romanian RABV cases from 2025.
