# Supplementary material for: Diagnostic and Phylogenetic Insights into a Human Rabies Virus Isolate from Romania
Source: Viruses. 2026 Apr 17;18(4):475. doi: 10.3390/v18040475 (PMC13120668; doi:10.3390/v18040475)
Supplement: Supplementary file 1 [file viruses-18-00475-s001.zip › viruses-4206373-SI.pdf]

Diagnostic and Phylogenetic Insights into a Human Rabies Virus Isolate from Romania

Vlad Vuta[1)], Maria Gradinaru[2)], Mihnea Hurmuzache[2)], Florica Bărbuceanu[1), 3)], Lenuta Zamfir[1)], Răzvan Moțiu[1)], Laura Schmid[4)], Dirk Höper[4)], Sten Calvelage[4)], Thomas Mueller[4)], Conrad M. Freuling[4)]

1) Institute for Diagnosis and Animal Health, Bucharest, Romania

2) Clinical Hospital of Infectious Diseases, Iasi, Romania

3) Faculty of Veterinary Medicine, Bucharest, Romania

4) Friedrich-Loeffler-Institute, Greifswald - Insel Riems, Germany

*) Corresponding authors: Conrad Freuling (Conrad.Freuling@fli.de)

Supplementary Material

Methos S1

Next-generation sequencing (NGS)

For each sample, ca. 1-2g of brain material was homogenized on an UPHO tissue homogenizer (Geneye) and 750 µl TRIzol added to 250 µl homogenate for inactivation. The TRIZol-sample mixture was processed with 200 µl chloroform followed by a ten-minute incubation at room temperature and subsequent centrifugation for ten minutes at 13.000 rounds per minute. RNA was extracted from the separated aqueous phase utilizing the RNAdvance Tissue Kit (Beckman Coulter) on a KingFisher Flex platform (Thermo Fisher Scientific) including a DNAse digestions step. Extracted and cleaned RNAs were quantified on an Implen N60 Nanophotometer. For the generation of double stranded cDNA, 350 ng of RNA were processed with the Super Script™ IV First Strand Synthesis System (Thermo Fisher Scientific) followed by a second strand synthesis with the NEBNext® Ultra II Non-Directional RNA Second Strand Synthesis Modul (E6111L, New England Biolabs) according to the manufactures instructions. The obtained cDNAs were cleaned with a 1.8-fold sample volume of Agencourt AMPure XP beads (Beckman Coulter) before fragmentation on a Covaris M220 Focused ultrasonicator (Covaris). Fragmented cDNAs were concentrated with AMPure XP beads to prepare the generation of Ion Torrent™ compatible libraries with the NEBNext® Fast DNA Library Prep Set for Ion Torrent™ (E6270L, New England Biolabs) in combination with IonXpress Barcode Adapters (Thermo Fisher Scientific). Following an AMPure XP bead-based size selection step, final libraries were quality checked on a Bioanalyzer

2100 (Agilent) utilizing the High Sensitivity DNA Chip Kit (Agilent) and quantified with the QIAseq Library Quant Assay Kit (Qiagen). Library pools were loaded on Ion 530™chips by an Ion Chef™ instrument (Thermo Fisher Scientific) and sequenced on an S5XL platform (Thermo Fisher Scientific) in 400 bp mode. To obtain complete RABV genome sequences, generated NGS datasets were initially mapped against the RABV reference OM203138 utilizing the 454 Sequencing System Software v3.0 (Roche) with an included quality trimming. The resulting alignment was visualized with Geneious Prime v2025.1.3 (Dotmatics) and the consensus of mapped reads extracted to perform a second mapping of the raw reads against the consensus. The final alignment was again inspected in Geneious Prime and the consensus corrected if necessary. The so obtained complete genomes were aligned with publicly available RABV reference sequences from East European RABV outbreaks (see Supplementary Table S1) as well as the RABV reference sequence NC_001542 (predefined outgroup) utilizing the multiple sequence alignment software MAFFT v7.490 [19,21] (automatic method selection). Phylogenetic analyses were performed with IQ-Tree v3.0.1 including best fit model selection by ModelFinder [22] and 100.000 ultrafast bootstraps. Tree annotation was realized with iTOL [23] v7.2.2 (European Molecular Biology Laboratory).

Table S1 Reference sequences selected from public databases for the genetic classification of Romanian RABV cases from 2025.

Accession	Sample Id	Country	Host	Year	Publication
MW177593	DR1351	Moldova	Cat	2016	[24]
MW177595	DR1017	Romania	Cat	2014	[24]
OL440112	DR1031	Romania	Cattle	2015	[24]
OL449092	DR1021	Romania	Wolf	2014	[24]
OL449093	DR1019	Romania	Fox	2014	[24]
OL449095	DR1025	Romania	Fox	2012	[24]
OL515137	DR1024	Romania	Fox	2012	[24]
OL515138	DR1026	Romania	Dog	2012	[24]
OL515139	DR1027	Romania	Deer	2012	[24]
OL515140	DR1035	Romania	Cattle	2012	[24]
OL515141	DR1333	Romania	Fox	2016	[24]
OL515150	DR1198	Moldova	Goat	2016	[24]
OM021440	DR1200	Moldova	Dog	2016	[24]
OM203138	DR1348	Romania	Fox	2016	[24]
OM203141	DR1345	Moldova	Cattle	2016	[24]
OM542185	2244061018L	Poland	Fox	2018	[25]
OM542186	2246060119L	Poland	Fox	2019	[25]
OM542187	2247060320L	Poland	Fox	2020	[25]
OM542188	2248060420L	Poland	Fox	2020	[25]
OM542189	2250060620L	Poland	Fox	2020	[25]
OM542190	2251060720L	Poland	Fox	2020	[25]
OM542191	2252181020P	Poland	Dog	2020	[25]
OM542192	2249180620L	Poland	Fox	2020	[25]
OM542193	2255140121L	Poland	Fox	2021	[25]
OM542194	2256140121L	Poland	Fox	2021	[25]
OM542195	2258140221L	Poland	Fox	2021	[25]
OM542196	025140601L	Poland	Fox	2001	[25]
OM542197	137141200L	Poland	Fox	2000	[25]
OM542198	915140604K	Poland	Cat	2004	[25]
OM542199	2266140421L	Poland	Fox	2021	[25]
OM542200	2276140521L	Poland	Fox	2021	[25]
OM542201	2277140521J	Poland	Dog	2021	[25]
OM542202	2259140321L	Poland	Fox	2021	[25]
OM542203	2283140621L	Poland	Fox	2021	[25]
OM542204	2254180121Zb	Poland	Cat	2021	[25]

MK598368	1322	Hungary	Fox	2014	Direct submission
MK598362	30856	Hungary	Cattle	2013	Direct submission
MK598352	15582	Hungary	Fox	2013	Direct submission
MK598356	25611	Hungary	Fox	2013	Direct submission
MK598354	24951	Hungary	Fox	2013	Direct submission
MK598372	6003	Hungary	Fox	2014	Direct submission
MK598381	22329	Hungary	Fox	2013	Direct submission
MK598393	14940	Hungary	Fox	2014	Direct submission
MK598385	26627	Hungary	Fox	2013	Direct submission
MK598380	21541	Hungary	Fox	2013	Direct submission
OQ544454	VB747-2022_Badger_Jablon_SK	Slovenia	Badger	2022	[26]
OQ544455	VB1071-2022_Dog_V.Slemence_SK	Slovenia	Dog	2022	[26]
OQ544456	VB1135-2022_Fox_Rovne_SK	Slovenia	Fox	2022	[26]
PV178704	VB_995-2024_fox_Michalovce_836915_Slovakia	Slovenia	Fox	2024	Direct submission

Tissue preparation and immunolabelling

Cerebral tissue was fixed with 4% paraformaldehyde (PFA) in phosphate-buffered saline (PBS) and sectioned into 100 µm slices using a vibratome (VT1200S, Leica Biosystems, Germany). Sample preparation and immunolabelling protocols were performed essentially as described previously [27], with minor modifications; the tissue-clearing step was omitted. Sections were dehydrated through an ascending methanol (MeOH) series in distilled water (aq. dest.) (20%, 40%, 60%, 80%, 100%; 10 min each), followed by delipidation in 66% dichloromethane (DCM)/33% MeOH for 3 h. Samples were washed twice in 100% MeOH (30 min each) and bleached overnight in 5% H_2O_2 in MeOH. Rehydration was carried out through a decreasing MeOH series in aq. dest. (80%, 60%, 40%, 20%, 0%; 10 min each). All subsequent steps were performed at 37°C. Tissue permeabilization was conducted overnight (0,2% Triton X-100/20% DMSO/0,3 M Glycine/NaN_3 0,02% in PBS). Samples were then blocked for 24 h [6% donkey serum/10% DMSO/0,02% Triton X-100/0,02% NaN_3 in PTwH (0.2% Tween-20/10 µg/mL heparin in PBS)]. For RABV detection, sections were incubated for 24h with a polyclonal rabbit serum against recombinant RABV P protein (P160-5; 1:3000) [28] diluted in 3% donkey serum/5% DMSO in PTwH. Samples were washed six times in PTwH (final wash overnight), then incubated 24h with the secondary antibody Donkey anti-Rabbit IgG (H+L), Alexa Fluor™ Plus 488 (Invitrogen Antibodies; A32790; RRID: AB_2762833; 1:500) and Hoechst 33324 (Invitrogen; H3570; 1:20.000) in PTwH with 3% donkey serum. A final series of six washes in PTwH was performed before mounting the sections on microscope slides using ibidi Mounting Medium (REF 50001).

Confocal laser scanning microscopy and image processing

Image stack was acquired with a stepsize of 0,3 µm using a Leica Stellaris 8 microscope equipped with HC PL APO CS2 63x/1.40 OIL objective, and the single slice with a HC PL APO 20x/0.75 IMM CORR CS2 objective using the Leica application suite (LAS X 4.7.0.28176) software and then processed in ImageJ (2.14.0/1.54p).

References

19. Orbanz, J.; Finke, S. Generation of recombinant European bat lyssavirus type 1 and inter-genotypic compatibility of lyssavirus genotype 1 and 5 antigenome promoters. *Arch. Virol.* **2010**, *155*, 1631–1641.
21. Zaeck, L.; Potratz, M.; Feruling, C.M.; Müller, T.; Finke, S. High-Resolution 3D Imaging of Rabies Virus Infection in Sol-vent-Cleared Brain Tissue. *J. Vis. Exp.* **2019**. doi:10.3791/59402.
22. Katoh, K.; Misawa, K.; Kuma, K.; Miyata, T. MAFFT: a novel method for rapid multiple sequence alignment based on fast Fourier transform. *Nucleic Acids Research*. **2002**, *30*, 3059–3066. doi:10.1093/nar/gkf436.
23. Katoh, K.; Standley, D.M. MAFFT multiple sequence alignment software version 7: improvements in performance and usa-bility. *Mol. Biol. Evol.* **2013**, *30*, 772–780. doi:10.1093/molbev/mst010.
24. Kalyaanamoorthy, S.; Minh, B.Q.; Wong, T.K.F.; Haeseler, A. von; Jermiin, L.S. ModelFinder: fast model selection for accurate phylogenetic estimates. *Nat. Methods.* **2017**, *14*, 587–589. doi:10.1038/nmeth.4285.
25. Ciccarelli, F.D.; Doerks, T.; Mering, C. von; Creevey, C.J.; Snel, B.; Bork, P. Toward automatic reconstruction of a highly re-solved tree of life. *Sci.* **2006**, *311*, 1283–1287. doi:10.1126/science.1123061.
26. Dascalu, M.A.; Daraban, F.; Rusu, O.R.; Tanase, O.I.; Velescu, E. Epidemiological statistics of rabies in animals in Moldova region, Romania, between 2010 and 2014. *J. Biotechnol.* **2015**, *208*, S35-S35. doi:10.1016/j.jbiotec.2015.06.097.
27. Smreczak, M.; Orłowska, A.; Trębas, P.; Stolarek, A.; Freuling, C.; Müller, T. Re-emergence of rabies in Mazowieckie Voi-vodeship, Poland, 2021. *Zoonoses Public Health.* **2023**, *70*, 111–116, doi:10.1111/zph.13005.
28. Robardet, E.; Smreczak, M.; Orlowska, A.; Malik, P.; Nándori, A.; Dirbáková, Z.; Jerg, S.; Rudoi, O.; Polupan, I.; Groza, O.; et al. Two Sylvatic Rabies Re-Emergences in Central-Eastern Europe over the 2021–2022 Period: An Unprecedented Situation in Recent Years. *Transbound. Emerg. Dis. 2023* **2023**, 1–9. doi:10.1155/2023/5589201.
